# Unravelling the Affinity of Alkali-Activated Fly Ash Cubic Foams towards Heavy Metals Sorption

**DOI:** 10.3390/ma15041453

**Published:** 2022-02-15

**Authors:** Ana P. F. Caetano, João Carvalheiras, Luciano Senff, Maria P. Seabra, Robert C. Pullar, João A. Labrincha, Rui M. Novais

**Affiliations:** 1 Department of Materials and Ceramic Engineering/CICECO-Aveiro Institute of Materials, University of Aveiro, Campus Universitário de Santiago, 3810-193 Aveiro, Portugal; anacaetano@ua.pt (A.P.F.C.); jcarvalheiras@ua.pt (J.C.); pseabra@ua.pt (M.P.S.); robertcarlyle.pullar@unive.it (R.C.P.); jal@ua.pt (J.A.L.); 2 Department of Mobility Engineering, Federal University of Santa Catarina (UFSC), Joinville 89219-600, SC, Brazil; lsenff@gmail.com; 3 Department of Molecular Sciences and Nanosystems (DSMN), Ca’ Foscari University of Venice, Scientific Campus, Via Torino 155, 30172 Venice Mestre, Italy

**Keywords:** geopolymer, adsorption, porosity, wastewater treatment, ion selectivity

## Abstract

In this work, alkali-activated fly ash-derived foams were produced at room temperature by direct foaming using aluminum powder. The 1 cm^3^ foams (cubes) were then evaluated as adsorbents to extract heavy metals from aqueous solutions. The foams’ selectivity towards lead, cadmium, zinc, and copper ions was evaluated in single, binary, and multicomponent ionic solutions. In the single ion assays, the foams showed much higher affinity towards lead, compared to the other heavy metals; at 10 ppm, the removal efficiency reached 91.9% for lead, 83.2% for cadmium, 74.6% for copper, and 64.6% for zinc. The greater selectivity for lead was also seen in the binary tests. The results showed that the presence of zinc is detrimental to cadmium and copper sorption, while for lead it mainly affects the sorption rate, but not the ultimate removal efficiency. In the multicomponent assays, the removal efficiency for all the heavy metals was lower than the values seen in the single ion tests. However, the superior affinity for lead was preserved. This study decreases the existing knowledge gap regarding the potential of alkali-activated materials to act as heavy metals adsorbents under different scenarios.

## 1. Introduction

The critical water scarcity scenario observed in many parts of the world has triggered the pursuit of new water sources [1]. The exploitation of industrial effluents might be an excellent approach to cope with the increasing demand for fresh water, provided that their hazardous components (e.g., heavy metals) are removed [2]. The presence of heavy metals in industrial wastewaters threatens human health and, consequently, hinders the reuse of these effluents as a source of clean water [3].

The interest in the use of alkali-activated materials (binders produced by the alkali activation of reactive solid precursors [4]) for water and wastewater treatment is recent, despite their obvious suitability to act as adsorbents [5,6], endowed by their intrinsic nano- and micro-porosity [7], and their negatively charged framework balanced by cations that can be exchanged for other cations [8] present in wastewaters [9,10]. Other technical advantages over benchmark adsorbents (e.g., activated carbons) are the possibility of being produced at room temperature, and the use of various waste streams or by-products as precursors [11,12,13,14].

In adsorption studies focusing on the use of alkali-activated materials, the use of pulverised materials (powders) is the conventional approach [15,16], similar to that of activated carbons. However, this strategy requires the use of a post-separation step, increasing the complexity and cost of wastewater treatment systems. The use of bulk-type alkali activated adsorbents, as opposed to powders, is much less explored, with only a few investigations performed to date [9]. The present study aims to decrease the knowledge gap regarding the use of cm-size alkali-activated adsorbents, and in addition shed light on the foams’ affinity towards various heavy metals. The affinity of bulk-type alkali activated sorbents for different heavy metals has been little studied, one of the rare exceptions being the study of Tang et al. [17]. Despite their interesting results, the selectivity of the sorbents for Pb^2+^, Cu^2+^, and Ca^2+^ was only evaluated using single component assays. In this study, the affinity of the fly ash-derived foams for Pb^2+^, Cd^2+^, Cu^2+^, and Zn^2+^ was evaluated in single, binary, and multicomponent solutions. To the best of our knowledge, this type of investigation has never been performed for cm-size biomass fly ash-derived alkali activated foams.

## 2. Materials and Methods

### 2.1. Materials

A blend of biomass fly ash waste and metakaolin (Argical™ M1200S, Univar^®^, Montreuil, France) was activated using a mixture of sodium silicate (SiO_2_/Na_2_O = 3.1, H_2_O = 62.1 wt.%, Quimialmel, Albergaria-a-Velha, Portugal) and sodium hydroxide solutions (8 M, ACS reagent, 98%; AkzoNobel, Lucerne, Switzerland). Aluminium powder (Expandit BE 1101, Grimm Metallpulver GmbH, Roth, Germany), coupled with a surfactant (Hotaspur OSB, Clariant, Barcelona, Spain), was used to generate gas bubbles into the paste creating voids in the hardened body.

Nitric acid 68% and lead (II) nitrate (>99%) were purchased from VWR Chemicals (Radnor, PA, USA). Cadmium Chloride (>99%), copper (II) nitrate trihydrate (>99%), and zinc nitrate hexahydrate (>98%) were purchased from Sigma-Aldrich Chemicals (Steinheim, Germany). The heavy metals stock solutions were prepared with deionised water.

### 2.2. Synthesis of the Adsorbent

The alkali-activated foams were produced using a composition developed by the authors [18,19] containing a 70/30 fly ash and metakaolin blend activated with a 75/25 sodium silicate/sodium hydroxide solution (in weight). The molarity of the sodium hydroxide solution (8 M) and the foaming agent amount (0.1 wt.% aluminium plus 0.05 wt.% surfactant) were selected following our previous findings [20] and considering both technical and economic reasons. After mixing, the paste was transferred to rubber moulds (1 cm^3^), their top surface covered with a plastic film, and the specimens were cured at room temperature (23 °C) for 1 day. After this period, the hardened samples were extracted from the moulds, and cured at room temperature for 28 days. Then, and prior to the adsorption tests, the foams were neutralised in 0.01 M HNO_3_ (Panreac 65%, ISO) for 1 h, and washed with distilled water to extract the unbonded alkalis from their structure [21,22] to avoid heavy metals precipitation.

### 2.3. Heavy Metals Sorption Tests

To evaluate the foams’ affinity towards heavy metals extraction, single, binary, and multicomponent ion adsorption tests were performed. In the single sorption tests, the heavy metal element (Pb^2+^, Cd^2+^, Zn^2+^, Cu^2+^), concentration (10–800 ppm), and the contact time (1–6 h) between the adsorbent and the ion solution was varied. It should be noted that the maximum contact time (6 h) was defined considering a previous study by the authors showing that within this period the alkali-activated foams can efficiently extract lead from aqueous solutions [20]. The heavy metals initial concentrations varied between 100–800 ppm: 4.83 × 10^−5^–3.86 × 10^−3^ mol/L for Pb^2+^, 8.90 × 10^−5^–7.12 × 10^−3^ mol/L for Cd^2+^, 1.53 × 10^−4^–1.22 × 10^−2^ mol/L for Zn^2+^, 1.57 × 10^−4^–1.26 × 10^−2^ mol/L for Cu^2+^).

Adsorption was performed at room temperature (23 °C) on 1 cm^3^ foams (as cubes), using 100 mL of solution, and at a fixed pH of 5. This pH value was selected considering previous investigations showing that the lead [20,23], cadmium [24], zinc [25], and copper [26,27] sorption is favoured at this value, but also to avoid metal hydroxide precipitation that might take place at higher pHs. After this assessment, binary and multicomponent sorption tests were performed at the optimised conditions (C_0_ = 10 ppm, contact time: 6 h) to evaluate the selectivity of the foams in bi-component and multi-component sorption systems. The heavy metals concentration was measured by inductively coupled plasma—optical emission spectroscopy (ICP-OES, Horiba JobinYvon, Activa M, Kyoto, Japan). Aliquots of 1 mL were taken from the solution. Two replicas were used, and the average result presented.

### 2.4. Materials Characterisation

The apparent density was determined by measuring the weight and volume of seventy specimens. The total porosity was then calculated as in [21], considering a true density of 2.37 g/cm^3^ [20]. The water uptake by the foams was measured upon 24 h immersion in distilled water. Three replicas were used, and the average data reported.

The Brunauer-Emmett-Teller (BET) technique was used to determine the specific surface area of the foams by N_2_ adsorption using a 5-point BET method on a Micromeritics Gemini 2380 surface area analyser (Norcross, GA, USA). Prior to the analysis, the foams were cut into small slices of approximately 0.1 g and outgassed at 150 °C for 12 h.

Zeta-potential measurements were assessed using a Zetasizer Nano ZS (Malvern, Worcestershire, UK). The sample was dispersed in deionised water and the measurement done at room temperature (~23 °C) using sodium hydroxide or hydrogen chloride solution to adjust the pH level. Three replicas were evaluated, and the average result presented.

Scanning electron microscopy (SEM) equipped with energy dispersive spectroscopy (Hitachi SU70, Tokyo, Japan, Bruker-EDS, Billerica, MA, USA) was used to analyse the samples’ microstructure.

Fourier transform infrared spectroscopy with attenuated total reflection (FTIR-ATR) measurements, before and after heavy metals sorption tests, were performed on a Brücker IFS FTIR spectrophotometer (Billerica, MA, USA) equipped with a single horizontal Golden Gate diamond ATR cell. The tests were carried out with 8 cm^−1^ resolution and 256 scans.

## 3. Results and Discussion

### 3.1. Physical Characterisation of the Bulk-Type Adsorbent

**Figure 1:**
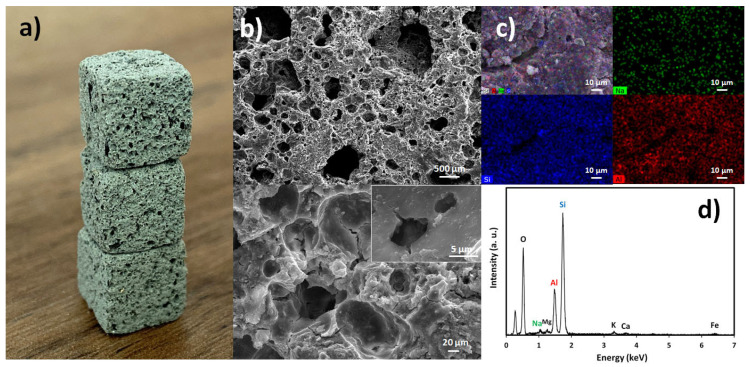
(**a**) Digital photograph and (**b**) SEM micrographs taken at two magnifications of the alkali-activated foams. (**c**) Shows the elemental mapping of Na, Si, and Al in the foams, and (**d**) the corresponding EDS spectrum.

A digital photograph of the bulk-type alkali-activated foams is provided in Figure 1a, while SEM micrographs illustrating the foams’ porosity are shown in Figure 1b. Alkali-activated materials are known to contain nano and micro-size pores [7,28], which can be coupled with macropores when foaming agents are added during their synthesis. Figure 1a shows the presence of a high number of large pores, up to the mm range, which are expected to favour the heavy metals uptake by the foams due to the higher number of active sites on their surface and interior compared to the unfoamed material. The SEM micrographs in Figure 1b, collected at various magnifications, further illustrate the hierarchical porosity of the produced sorbent. The foams’ total porosity reaches 77.8% (see Table 1) and their bulk density is 0.53 g/cm^3^. These values slightly differ from those recently reported by the authors (84.0% total porosity and 0.38 g/cm^3^) [20], despite the fact that the same recipe has been used in their synthesis. The explanation for the higher apparent density observed in the present study is attributed to the modification of the foams’ synthesis protocol, particularly the geometry of the mould. Here, the foamed slurry was directly cast in 1 cm^3^ rubber moulds, while in the previous work the paste was poured into 256 cm^3^ metallic moulds. The geometry of the mould is known to affect the expansion kinetics [29,30]. For example, the height and volume of the mold will influence the gas path and pressure on the paste, and thus the expansion of the slurry.

The water absorption of the foams (~73%) is also much lower than the value previously reported (131%) [20], indicating that the form of the pores was changed as well. The connectivity of the pores is a key feature when dealing with heavy metals adsorption, and these results show that the use of larger size moulds is a preferable option, enabling a greater expansion of the slurry and an increase in the volume of open pores. Despite this, the current approach is much simpler as the foams can be directly used for heavy metals adsorption, avoiding the need for additional processing steps (e.g., cutting), as in [20]. Moreover, the specific surface area of the foams (50.4 ± 0.5 m^2^/g), included in Table 1, is similar to those reported for other bulk-type alkali-activated materials [17,20,31], and this endows their use as adsorbent.

The physical properties of alkali-activated foams prepared by direct foaming can be tuned by the nature and amount of the foaming agent [32], but also using different surfactants [33]. However, it should be noted that the optimization of the foam’s physical properties is beyond the scope of the present study as the main objective was to evaluate the affinity towards different heavy metals. Nevertheless, the bulk density here reported is similar to that seen when using hydrogen peroxide (0.556 g/cm^3^), but the foams showed lower porosity (63.1%) compared to the value here reported (see Table 1) [34]. Ducman and Korat reported slightly higher density when using 0.13 wt.% Al (0.64 g/cm^3^) or 1.1 wt.% H_2_O_2_ (0.61 g/cm^3^) [32], while much higher values were observed by Masi et al. when using Al powder ranging from 0.94 to 1.42 g/cm^3^ [35]. Recently, much lower densities have been reported when coupling H_2_O_2_ with a cationic surfactant (cetyltrimethyl ammonium bromide) (as low as 0.25 g/cm^3^) [33], and this suggests that the properties of the fly ash foams can be further optimized to increase their porosity and specific surface area.

Figure 1c shows EDS maps for silicon, aluminium, and sodium collected from the surface of the foam, while Figure 1d presents the corresponding EDS spectrum. The EDS maps show a rather similar silicon and aluminium distribution within the samples, and a much lower content of sodium, proving that the neutralization step (see Experimental Section for details) was successful.

**Figure 2:**
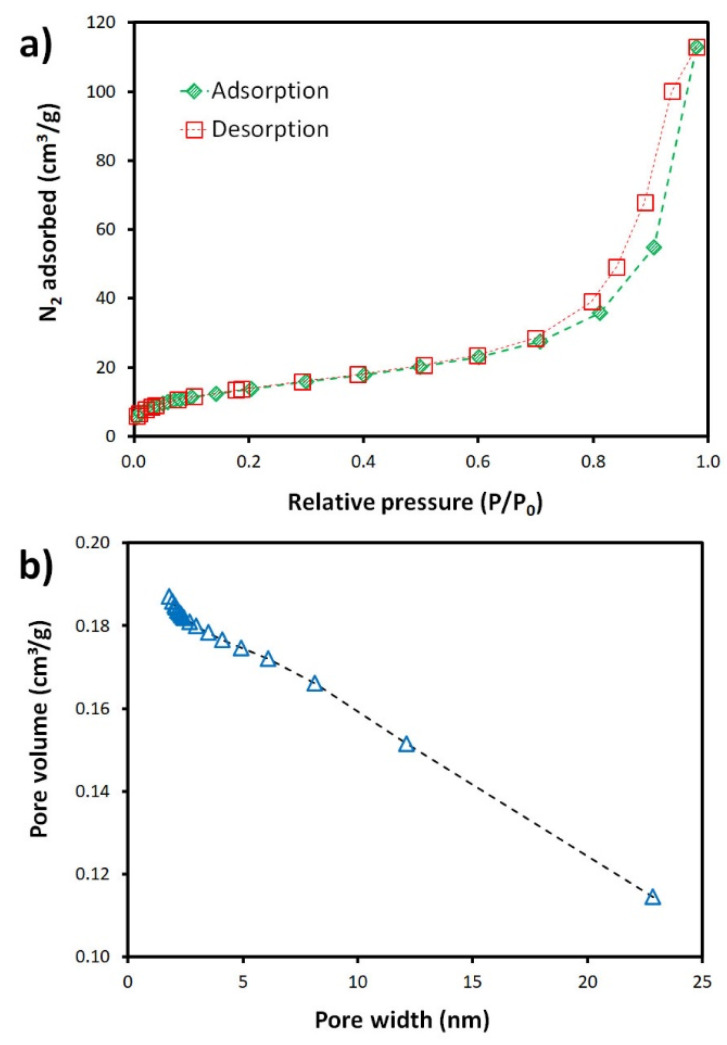
(**a**) N_2_ adsorption and desorption isotherms and (**b**) cumulative pore volume of the sorbent.

The N_2_ adsorption and desorption isotherms of the cm-size foam, measured at 77 K, are presented in Figure 2. The foam exhibits a type IV isotherm, typical of mesoporous adsorbents. At low pressure, these materials show a fairly poor N_2_ sorption, but higher relative pressures promote a significant increase in the N_2_ sorption volume. At low relative pressure, monolayer adsorption takes place in the micropores, and then multilayer sorption begins filling the mesopores with capillary condensation giving rise to the hysteresis loop [36] seen in Figure 2a. Figure 2b presents the cumulative pore volume of the foam, showing that it contains mostly mesopores (2–50 nm), the micropores >2 nm diameter corresponding to roughly 2% of the total pore volume.

**Figure 3:**
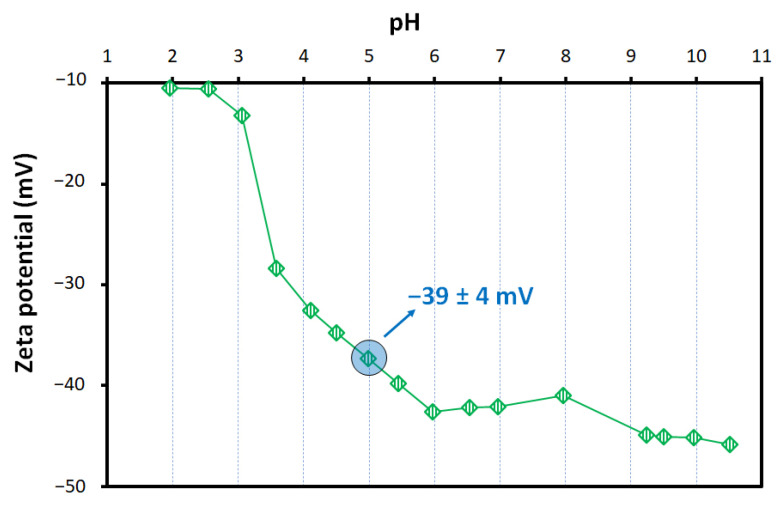
Zeta potential fluctuation of the alkali-activated foam with pH. The average zeta potential from three replicas at the pH of the adsorption tests (pH = 5) is shown.

The zeta potential of the alkali-activated fly ash was measured at pH values ranging from 2 to 10.5, and the results are presented in Figure 3. The results show that the foams have a negatively charged framework regardless of the pH. Nevertheless, their surface charge is pH-dependent, higher pHs decreasing the surface charge density. This feature is particularly relevant in the low pH range, between 2 and 6, while at higher pH values, the surface charge fluctuation is minor but maintains a decreasing trend. In this investigation, the adsorption tests were performed at fixed pH (pH = 5), as detailed in the experimental part (see Section 2.3). At this pH, the surface charge of the alkali-activated fly ash is −39 ± 4 mV, enabling the use of the foams to extract cationic species from wastewaters. The negative zeta potential seen for the foams is attributed to the negative charge of the alkali-activated materials [10]. Their framework is composed by AlO_4_ and SiO_4_ units linked alternatively by shared oxygen atoms [37]. The oxygen tetrahedra containing the Al^3+^ ions has a negative charge, which is balanced by cations such as Na^+^ [38], and this explains the negative zeta potential. The results shown in Figure 2 are in line with previous studies on the topic [39,40].

### 3.2. Heavy Metals Adsorption Tests

#### Single Component

**Figure 4:**
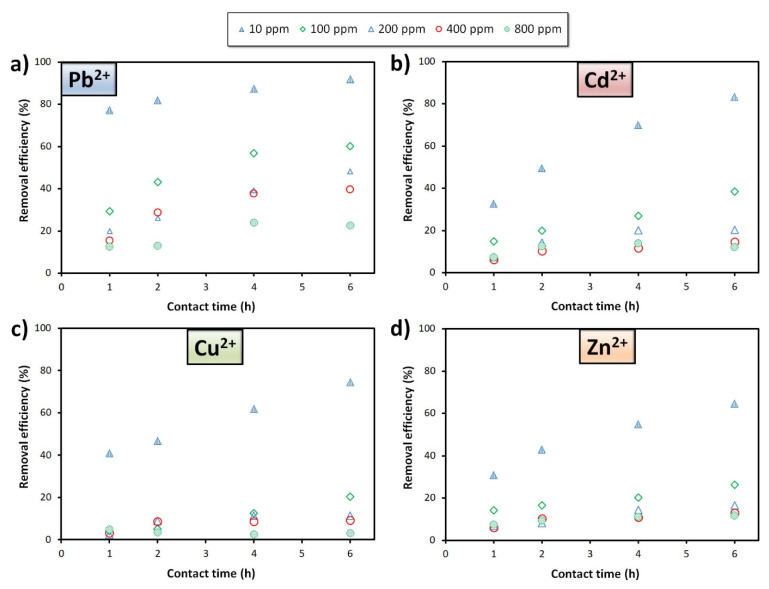
Influence of the pollutant initial concentration on the removal efficiency of (**a**) Pb^2+^, (**b**) Cd^2+^, (**c**) Cu^2+^, and (**d**) Zn^2+^ by the alkali-activated fly ash (pH = 5, volume: 100 mL).

**Figure 5:**
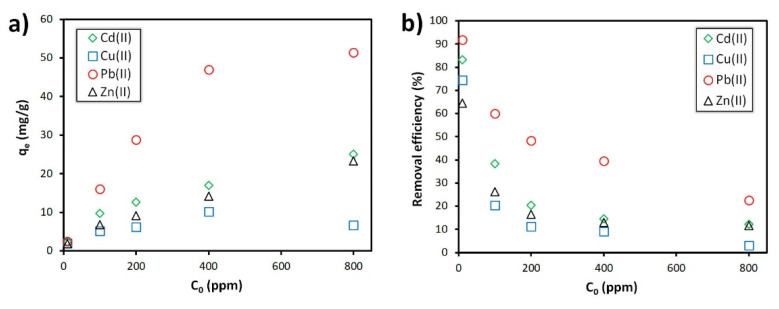
Influence of pollutant concentration on (**a**) the uptake and (**b**) removal efficiency of heavy metals by the alkali-activated fly ash (pH = 5, contact time: 6 h).

The influence of contact time and heavy metal initial concentration on the foams’ removal efficiency for the various heavy metals is presented in Figure 4. The results show that the use of longer contact times (6 h) favour the heavy metals’ extraction, this being particularly visible when the pollutant concentration is below ≤100 ppm. For example, at 100 ppm, the lead removal efficiency increases by a factor of 2, going from 29.4% (1 h) to 60.1% (6 h). The pollutant initial concentration also plays a major role, the use of higher pollutant concentrations negatively affecting the foams’ removal efficiency. For example, the lead removal efficiency (6 h contact time) drops from 91.9% ([Pb^2+^]_0_ = 10 ppm) to 22.5% ([Pb^2+^]_0_ = 800 ppm). Interestingly, the removal efficiency is highly dependent on the nature of the heavy metals, much higher values being observed when using lead compared to the other ions studied (Cd^2+^, Cu^2+^, Zn^2+^), and this suggests a different affinity of the foams towards the various heavy metals. This feature is better illustrated in Figure 5, where the foams’ uptake and removal efficiency for the various heavy metals is directly compared. As can be seen, the foams’ removal capacity sharply increases when the pollutant concentration rises, and this is despite the observed drop in the removal efficiency (see Figure 5b). In the case of lead, the uptake increases substantially, reaching an impressive value of 51.4 mg/g at C_0_ = 800 ppm, this being 20 times greater than the value observed for a 10 ppm solution (2.5 mg/g). In the single component assays, the foams showed similar affinity towards zinc and cadmium ions, the maximum uptake being 23.3 and 25.0 mg/g, respectively. The poorest adsorption was reached with copper (II), the maximum uptake being 10.2 mg/g at C_0_ = 400 ppm, decreasing to 6.7 mg/g at 800 ppm.

The comparison between the uptake reached at the highest pollutant concentration (800 ppm) shows that the Pb^2+^ ions extraction capacity by the foams is two times greater than that seen for both cadmium and zinc ions, and is 7.7 times higher compared to Cu^2+^. These results show that the fly ash-derived foams show the following selectivity: Pb^2+^ > Cd^2+^ > Zn^2+^ > Cu^2+^. The foams’ affinity towards the different heavy metals is dependent on several factors, including the hydrated ionic radius and the hydration enthalpy. Table 2 shows that Pb^2+^ ions present the lower hydrated ionic radius coupled with the highest hydration enthalpy which favours their extraction by the foams, this being the reason for their much higher removal capacity compared to the other studied cations. The slightly higher uptake observed for the Cd^2+^ ions compared to Zn^2+^ can be attributed to the higher hydration enthalpy of the former. As for Cu^2+^, it has a hydration enthalpy similar, but slightly superior, to Zn^2+^, and also with a smaller hydrated radius, which should result in a higher removal capacity. However, the results presented in Figure 5 show that the adsorption of Cu (II) ions by the foams is always smaller, and this is regardless of the initial pollutant concentration. Nevertheless, the differences between Cu^2+^ and the Zn^2+^ sorption are intensified by the use of higher pollutant concentrations. The tendency seen for the heavy metals affinity/selectivity (Pb^2+^ > Cd^2+^ > Zn^2+^ > Cu^2+^) by the foams is in line with previous results reported in the literature (e.g., zeolite-containing geopolymers [15] and metakaolin-based geopolymers [27]). However, in [41], a higher selectivity was seen for Cu^2+^ ions compared to the Cd^2+^, but similar to our findings, a much higher lead (II) sorption was observed compared to Cu (II) and the Cd (II).

**Figure 6:**
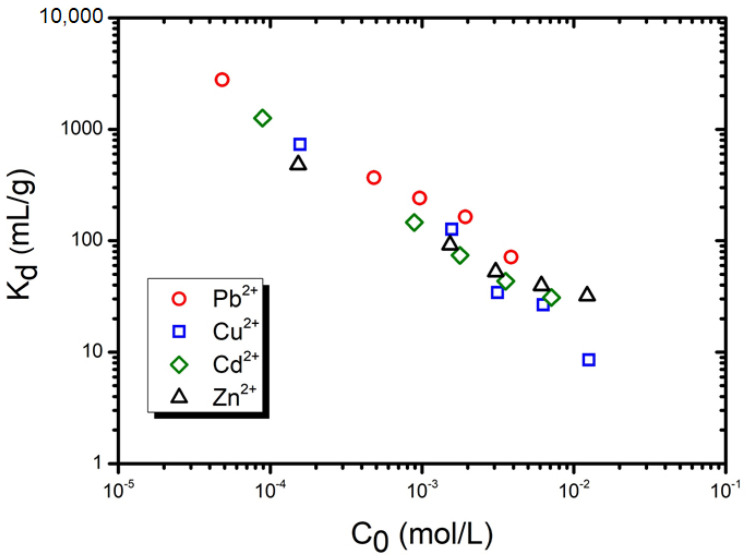
Fluctuation of the distribution coefficient as a function of the heavy metal initial concentration.

To further characterize the sorbents capacity for the different heavy metals, the distribution coefficient (K_d_ = q_e_/C_e_) [43] was determined for the highest contact time (6 h); the results are shown in Figure 6. The highest values were seen in the lowest concentration (10 ppm), varying between 482.1 mL/g for Zn^2+^ to 2775.3 mL/g for Pb^2^^+^—732.1 mL/g for Cu^2+^ and 1259.5 mL/g for Cd^2+^. Taking lead as an example, this means that the foams can treat 2.8 L/g. Nevertheless, it should be noted that the foams did not reach the sorption equilibrium, and this suggests that higher values can be expected when extending the sorption period. The results also show that an increase in the pollutant initial concentration induces a decrease in the distribution coefficient, in line with previous reports for other alkali-activated materials [43,44].

**Figure 7:**
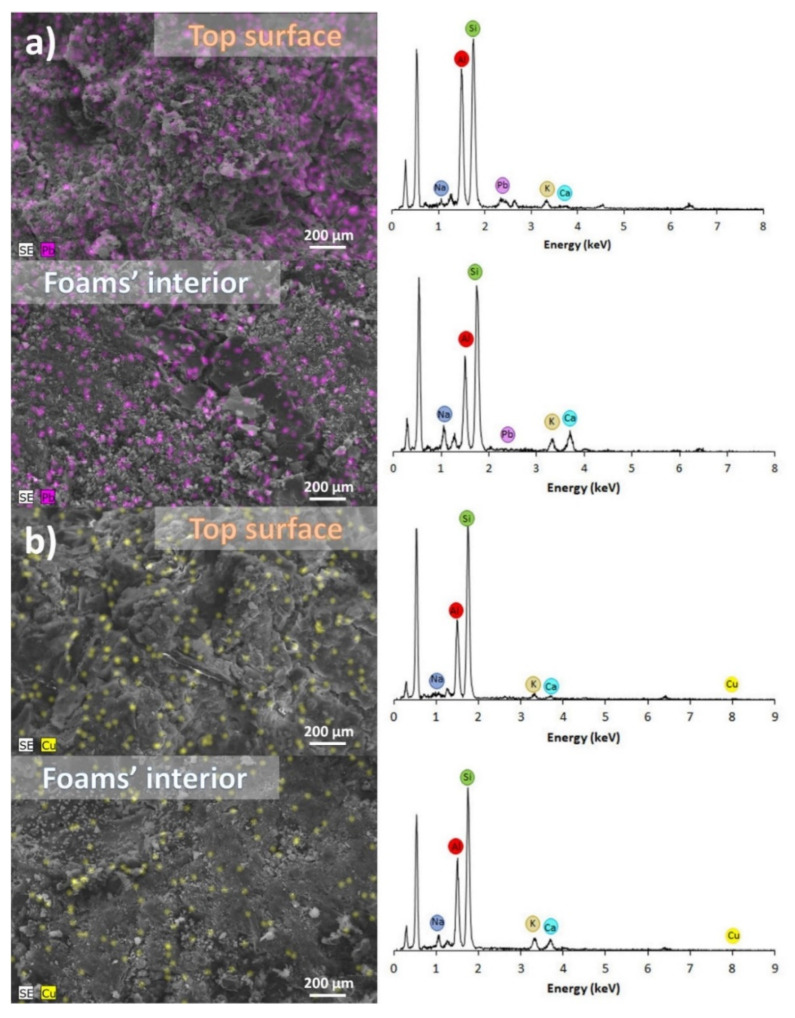
EDS maps (500× magnification) and spectra of the foams surface and inner part after the adsorption tests with: (**a**) lead and (**b**) copper (pH = 5, contact time: 6 h, [C0] = 800 ppm).

The heavy metals’ adsorption by the alkali-activated foams was further evaluated by SEM/EDS analysis, and representative EDS maps and spectra collected from the foams’ surface and inner part are presented in Figure 7a for lead and copper (Figure 7b). It should be noted that this technique can only provide a semi-quantitative analysis, but despite this, EDS results show a much higher adsorption of lead on the samples’ surface (5.0 wt.%) compared to their inner part (0.9 wt.%), suggesting that sorption is mostly occurring in the specimens’ surface. The same trend is seen for copper, but in this case much lower amounts of copper are detected (1.5 wt.% on the surface vs. 0.9 wt.% in the inner part of the foams), in line with the lower uptake seen for copper compared to lead ions, as can be seen by the results provided in Figure 5. There are two possible explanations for the differences seen in the adsorption of the pollutants in the different parts of the specimens (surface vs. inner part): (i) the low sorption time used in this investigation (6 h) limits the diffusion of the heavy metal cations into the specimens. Previous studies have shown that longer sorption times favour the diffusion of a pollutant into bulk-type adsorbents [45], resulting in higher uptakes [46]; and/or (ii) insufficient open porosity in the foams hindering the access of the heavy metals to active sites existing in the interior of the foams. An increase in the samples’ porosity is known to enhance the heavy metals’ sorption by the foams [20], and this strategy can be used to further enhance the samples’ performance.

**Figure 8:**
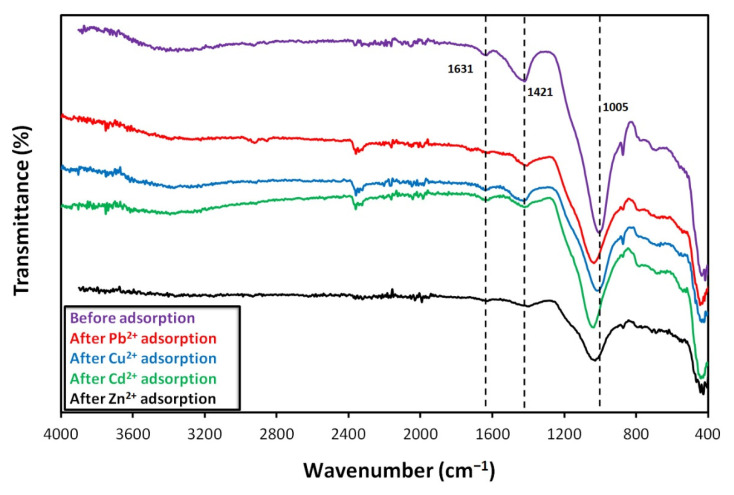
FTIR spectra of the adsorbent before and after heavy metals adsorption tests (pH = 5, contact time: 6 h, [C_0_] = 800 ppm).

Figure 8 presents the FTIR spectra of the alkali-activated fly ash foams before and after the heavy metals’ adsorption tests. Prior to the adsorption tests, the spectrum of the foams shows the characteristic band of alkali-activated materials at ~1000 cm^−1^ corresponding to the asymmetric stretching vibrations of Si–O–Si and Si–O–Al [47,48]. The peak at 1421 cm^−1^ is attributed to stretching vibrations of O–C–O bonds resulting from carbonation, while the peak at 1631 cm^−1^ is due to the bending vibration of H–O–H. After adsorption, the main bands (Si–O–Si and Si–O–Al) shift to a higher wavenumber, the extent of the shift being dependent on the heavy metal element. This feature has been associated with the presence of heavy metals [20,25] promoting changes in the local environment of the alkali-activated materials framework, as charge balancing cations (e.g., Na^+^ and K^+^) are partially exchanged [49]. Another feature is the decrease in the intensity of the peak at 1421 cm^−1^, suggesting that the reaction between the residual sodium in the sorbents and the atmospheric CO_2_ is now minor. The intensity of the peak at 1631 cm^−1^ also slightly decreases possibly due to the additional drying step performed to the specimens prior to this analysis (12 h at 100 °C).

Previous literature suggests that ion exchange is the main adsorption mechanism behind the extraction of heavy metals by alkali-activated materials [10,20]. Nevertheless, the chemical interaction between the heavy metals and the functional groups in the sorbents surface [50,51] has also been reported. Future work should be carried out to identify the main sorption mechanism.

As mentioned above, the main objective of the present study is the evaluation of the foams’ affinity towards the different heavy metals, attempting to decrease the existing knowledge gap. In this context, the optimisation of the foams’ performance (e.g., by increasing their surface area) was not considered. Nevertheless, the comparison with existing literature shows the potential of cm-size fly ash-derived sorbents: the maximum lead uptake (51.4 mg/g) is amongst the highest ever reported for bulk-type (not powders) alkali-activated materials [9], being higher than those reported for cylindrical discs [21], granules [22], geopolymer-supported zeolites [45], and spheres [17], and being only inferior to our previous study [20]. As for Zn^2+^, the maximum removal capacity (23.3 mg/g) is 3.1 times higher than that reported when using granules (7.4 mg/g) [22]. In obverse, the removal capacity of Cu^2+^ (10.2 mg/g) is much lower than that reported for metakaolin-based (35.5 mg/g) [17] and geopolymer/alginate spheres (60.8 mg/g) [52], despite being similar to the values show by zeolitic-tuff geopolymers (7.8 mg/g) [53]. The maximum Cd^2+^ uptake shown by the foams (25 mg/g) is much higher than that reported for pulverized alkali-activated metakaolin (3 mg/g [54]), being similar to the value seen with pulverized zeolite-based alkali-activated materials (26.2 mg/g). The reuse of the foams in multiple sorption cycles will be evaluated in a follow-up study. Nevertheless, literature shows that the use of mild acidic solutions [20] allow the regeneration of the sorbents. Future work will also evaluate the incorporation of the exhausted bodies as aggregates in the production of alkali-activated mortars to reach a zero-waste approach.

### 3.3. Binary and Multicomponent Assays

To provide additional insights on the foam’s affinity towards distinct heavy metals, additional sorption tests were performed. First, binary assays containing six different combinations of the heavy metals (Pb^2+^ and Cd^2+^, Pb^2+^ and Zn^2+^, Pb^2+^ and Cu^2+^, Cu^2+^ and Cd^2+^, Cu^2+^ and Zn^2+^, Cd^2+^ and Zn^2+^) were studied, and then a multicomponent system containing all the heavy metals (Pb^2+^, Cu^2+^, Zn^2+^, Cd^2+^) was also evaluated. Considering the results shown in the previous section, the heavy metals’ initial concentration in these tests was fixed at 10 ppm, as this ensured the highest removal efficiency in the single component assays (see Figure 4 and Figure 5).

**Figure 9:**
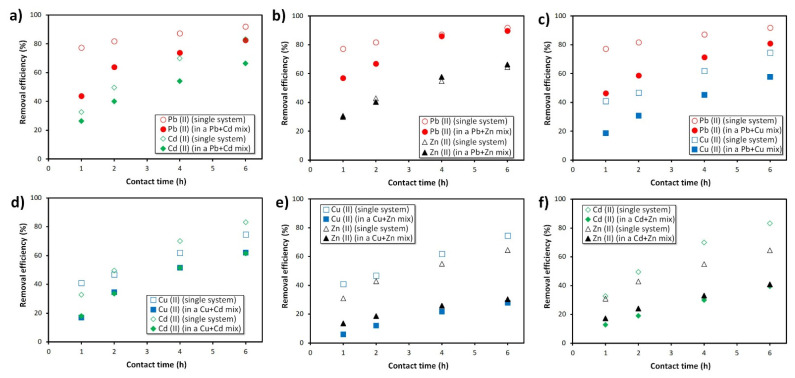
Influence of coexisting heavy metals on their removal efficiency in binary systems: (**a**) Pb^2+^ and Cd^2+^, (**b**) Pb^2+^ and Zn^2+^, (**c**) Pb^2+^ and Cu^2+^, (**d**) Cu^2+^ and Cd^2+^, (**e**) Cu^2+^ and Zn^2+^, and (**f**) Cd^2+^ and Zn^2+^ by the alkali-activated fly ash (C_0_ = 10 ppm, pH = 5, volume: 100 mL). Note: the removal efficiency obtained in the single system tests was included in all charts for comparison.

Figure 9 shows that the removal efficiency of lead (II) ions is affected by the presence of both Cd^2+^ and Cu^2+^, dropping from 91.9% (single system test) to 82.7% and to 80.9% in the presence of Cd^2+^ and Cu^2+^, respectively. The same decreasing trend is also seen for Cd^2+^ and Cu^2+^, both dropping by ~17%—Cd^2+^ from 83.2% to 66.4% and Cu^2+^ from 74.6% to 57.8%. These results show that the decrease in removal efficiency for cadmium and copper is much higher than that seen for lead in binary systems, suggesting a greater affinity of the fly ash-derived foams for lead.

The presence of Zn^2+^ (see Figure 9b) is found to decrease the Pb^2+^ sorption rate; within the first hour, the lead removal efficiency is roughly 20% lower compared to the single component assay. Nevertheless, the differences are mostly lessened as the sorption time increases. In fact, after 6 h, the lead removal efficiency in the binary system reaches ~90%, while for zinc the value reaches 66.4%, this being slightly higher than the value seen when only Zn^2+^ ions were in solution (64.6%). This feature demonstrates that the lead and the zinc adsorption by the foams is not substantially affected by the presence of the other heavy metal. On the contrary, the presence of Cd^2+^ and Cu^2+^ strongly affects the Zn^2+^ sorption. Figure 9e shows a twofold decrease in zinc removal efficiency (from 64.6% to 30.5%) in the presence of copper, while cadmium (Figure 9f) induces a lower, but significant, drop to 40.9%. The system containing both cadmium and zinc (Figure 9d) follows the same decreasing trend in the removal efficiency compared to the single assays, which is associated with the competition between the heavy metal ions for the active sites available in the adsorbent. Nevertheless, the removal efficiency for both ions in the binary system remains above 60%, much higher than the values seen in the presence of Zn^2+^.

**Figure 10:**
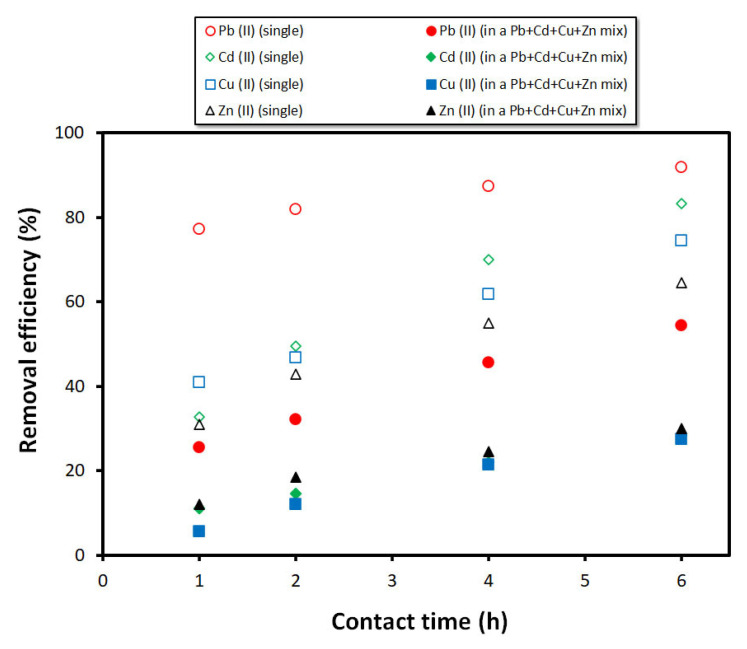
Influence of coexisting heavy metals on their removal efficiency in a multicomponent system (C_0_ = 10 ppm, pH = 5, volume: 100 mL).

Figure 10 presents the heavy metals removal efficiency in a multicomponent assay attempting to mimic real effluents, which can be very complex systems and where the competition between various heavy metals or pollutants is to be expected. The results show a major decrease in the removal efficiency of all of the studied heavy metals compared to the single element assays, ranging in decreases from 34.6% for Zn^2+^ to 54.7% for Cd^2+^. As for Pb^2+^, the removal efficiency decreased 37.5% (from 91.9% to 54.4%), while for Cu^2+^ the decrease was 47.1% (from 74.6% to 27.5%). It is interesting to note that the Zn^2+^ removal efficiency in this multicomponent assay is between the values seen in the binary systems composed of Zn^2+^/Cu^2+^ (30.5%, see Figure 9e) and Zn^2+^/Cd^2+^ (40.9%, see Figure 9f), while the copper (II) removal efficiency is virtually identical to the Zn^2+^/Cu^2+^ test (27.8%, see Figure 9e). These results suggest that the presence of zinc (II) in solution not only hinders, but also seems to determine the copper (II) and cadmium (II) extraction ability of the foams. Figure 10 also shows a higher selectivity of the foams towards the lead ions, in line with the results observed for the single component tests. However, in a multicomponent system, similar affinity by the foams towards copper (II), cadmium (II), and zinc (II) is observed, at least under the conditions used (e.g., C_0_ = 10 ppm). These results provide new insights into the foams’ affinity towards these heavy metals when dealing with multicomponent systems, which are closer to the sorbents’ real-life operation, and might contribute to the wider use of this technology.

## 4. Conclusions

In this work, and for the first time, the affinity of bulk-type (not powders) fly ash-derived foams for Pb^2+^, Cd^2+^, Cu^2+^, and Zn^2+^ was evaluated in single, binary, and multicomponent ionic solutions. Under the single ion tests, the foams showed the following affinity: Pb^2+^ > Cd^2+^ > Zn^2+^ > Cu^2+^, which is associated with the distinct hydrated ionic radius and hydration enthalpy of the studied heavy metals. In the highest pollutant concentration (800 ppm), the lead extraction capacity was 51.4 mg/g, which is amongst the highest values ever reported for bulk-type alkali-activated materials. In the same experimental conditions, zinc (23.3 mg/g) and cadmium (25.0 mg/g) showed similar removal capacity to one-another, while a much lower value was observed for copper (6.7 mg/g). In the binary systems, the superior affinity of the foams towards the lead ions was preserved, and this was regardless of the presence of competing ions. Nevertheless, the presence of cadmium and copper hindered the lead extraction ability. Interestingly, the presence of zinc in the binary test delayed lead sorption, but not the equilibrium uptake, as longer sorption times lessened the differences. In the multicomponent solution, the removal efficiency of all heavy metals was lower than the values seen in the single ion systems, but nevertheless, the higher selectivity for lead was still observed. In addition, the results suggest that zinc plays a major role in hindering cadmium and copper adsorption by the foams.

## Figures and Tables

**Table 1:** Physical properties of the alkali-activated foams.

Property	Average Value ± Standard Deviation
Bulk density (g/cm^3^)	0.53 ± 0.04
Water absorption (wt.%)	73 ± 6
Total porosity (%)	77.8 ± 1.7
Specific surface area (m^2^/g)	50.4 ± 0.5
Total pore volume (cm^3^/g)	0.191 ± 0.08

**Table 2:** Physicochemical parameters with regard to Pb, Cd, Cu, and Zn. Reprinted from [42] with permission from Elsevier.

Ions	Hydrated Ionic Radius (Å)	Electronegativity	Hydration Enthalpy (kJ/mol)	Hydrolysis Constant logK_MOH_
Pb^2+^	4.01	2.33	−1479.9	−7.71
Cd^2+^	4.26	1.69	−1807	−10.8
Cu^2+^	4.19	1.90	−2009	−8.00
Zn^2+^	4.30	1.65	−2046	−8.96

## Data Availability

Data sharing is not applicable to this article.
